# Factors associated with non-fusion after direct pars repair of lumbar spondylolysis with pedicle screw and lamina hook: a clinical and CT-assessed study

**DOI:** 10.1186/s12891-024-07252-0

**Published:** 2024-02-17

**Authors:** Xinhu Guo, Zhuofu Li, Zhaoqing Guo, Weishi Li

**Affiliations:** 1 Department of Orthopaedics, Peking University Third Hospital, 49 North Garden Road, Haidian District, Beijing, 100191 China; 2 Beijing Key Laboratory of Spinal Disease Research, Beijing, China; 3 Engineering Research Center of Bone and Joint Precision Medicine, Ministry of Education, Beijing, China

**Keywords:** Lumbar spondylolysis, Pars repair, Pedicle screw and lamina hook, Non-fusion

## Abstract

**Background:**

Pedicle screw and lamina hook (PSLH) technique is an effective and popular method for direct pars repair of lumbar spondylolysis. There is a lack of studies to explore factors that may influence the healing of spondylolysis after direct pars repair surgery. The present study aimed to investigate the factors associated with non-fusion after direct pars repair of lumbar spondylolysis with PSLH technique.

**Methods:**

A total of 55 subjects (average age 21.1 ± 6.3 years, a total of 120 pars defects) diagnosed with symptomatic spondylolysis and underwent pars repair surgery with PSLH were followed up and their clinical data were analyzed. Subjects were divided into a non-fusion group and fusion group according to whether the pars defect had bony fusion at last follow-up assessed by CT. Radiographic data, data related to spondylolysis and clinical outcomes were collected and compared between groups.

**Results:**

The mean follow-up time of the 55 patients was 24.8 ± 12.0 (12–64) months. Among the 120 pars defects, 101 defects were successfully fused and 19 were not fused according to CT. The fusion rate was 84.2%. Multivariable logistic regression analysis showed the factors correlated with non-fusion after pars repair surgery: whether the spondylolysis segment was associated with spina bifida occulta (SBO) (*P* = 0.001), stage of the defect (*P* = 0.047), width of the defect (*P* = 0.002), and disc degeneration (*P* = 0.014).

**Conclusion:**

Direct pars repair by PSHL is a reliable treatment for lumbar spondylolysis with a fusion rate of 84.2%. Association with SBO of the spondylolysis segment, a terminal stage of the defect, a wider defect gap, and grade III disc degeneration may be factors associated with non-fusion after direct pars repair of lumbar spondylolysis with PLSH. Non-fusion patients after pars repair appear to have worse clinical results compared to fusion patients.

## Background

Lumbar spondylolysis refers to a bony defect (or stress reaction) in the pars interarticular of the lumbar vertebra, which can be unilateral or bilateral [1]. In the general pediatric population, its prevalence ranges from 3 to 7% and increases up to 13 − 47% in pediatric patients presenting with low back pain [1, 2]. Conservative treatment is the gold standard, with good clinical outcomes for most patients [1–4].

Direct pars repair surgery is advised in symptomatic spondylolysis patients who fail to respond to conservative treatment or when there is progression to spondylolisthesis [1–5]. Among the several types of direct repair, the pedicle screw and lamina hook (PSLH) technique is believed to be an effective and popular method [2–4, 6]. Meta-analysis comparing different pars repair techniques showed that the pedicle screw-based pars repair had the highest fusion and lowest complication rates, which were 90.21% and 12.8%, respectively [6]. However, in the literature, the reported fusion rates of the PSLH technique vary widely, from 63.2 to 100% [6–13]. The following reasons may explain the discrepancy among the studies: (1) Most studies were retrospective studies with a small sample size; (2) Surgical indications for repair surgery of spondylolysis vary in different studies; (3) Age, the degree of disc degeneration, slippage, and follow-up time may be different among different studies, and the evaluation criteria of successful fusion may also be different. Although some studies have suggested that age and the degree of disc degeneration may influence healing of the defect after surgery, the causes of nonunion were not analyzed in enough detail to accurately describe the related factors for postoperative nonunion [8, 9]. For conservative treatment, the reported factors that may influence the healing of spondylolysis include the stage of the defect, the vertebral level of the defect, slipping of the affected vertebra, etc. [14–16]. However, for surgical treatment of spondylolysis, there is a lack of such studies. The purpose of this study was to investigate the factors associated with non-fusion after direct pars repair of lumbar spondylolysis with PSLH.

## Methods

### Patients

This is a retrospective study. Between January 2011 and August 2021, a total of 78 patients underwent direct pars repair surgery with PSLH in a tertiary hospital. The inclusion criteria were: (1) a diagnose of symptomatic spondylolysis with no more than grade I spondylolisthesis; (2) underwent pars repair surgery with PSLH technique; (3) age ≤ 35 years; (4) availability of lumbar CT at one-year follow-up or more to assess whether the defect had bony fusion. Patients who underwent previous spinal surgery were excluded. This study was reviewed and approved by the hospital’s ethics committee. According to the inclusion criteria, a total of 55 patients with a follow-up rate of 70.5% were included in this study.

### Radiographic data

General information was collected, including age, sex, and BMI. All patients had a whole-spine X-ray, lumbar spine X-ray, lumbar CT and MRI before surgery. The radiological data included: (1) lumbar lordosis (LL), defined as the angle between the upper endplate of L1 and the upper endplate of S1; (2) Pelvic incidence (PI), the angle between a line joining the center of the upper endplate of S1 to the axis of the femoral heads and a line perpendicular to the upper endplate of S1; (3) Sacral slope (SS), defined as the angle between the upper endplate and the horizontal line; (4) Pelvic tilt (PT), the angle between the vertical line and a line drawn from the center of the upper endplate of S1 to the axis of the femoral heads.

### Evaluation of spondylolysis

**Fig. 1:**
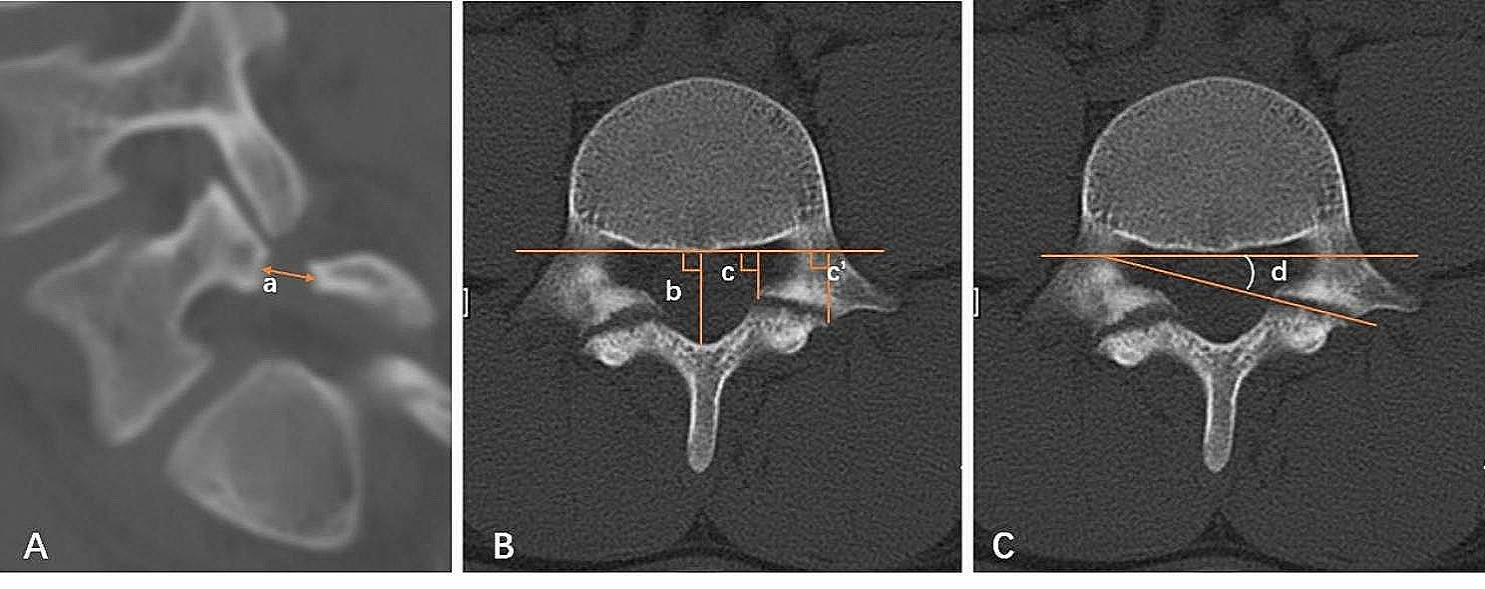
The width of the defect (segment a) is measured on reconstructed sagittal CT image in the middle of the pars interarticularis **(A)**. The distance of the defect to the vertebral body is calculated on axial CT image by the following formula: distance = (c + c’)/2b **(B)**. Angle of the defect (angle d) is the angle between the line of the posterior margin of the vertebral body and the line of the defect on the axial CT image **(C)**

**Fig. 2:**
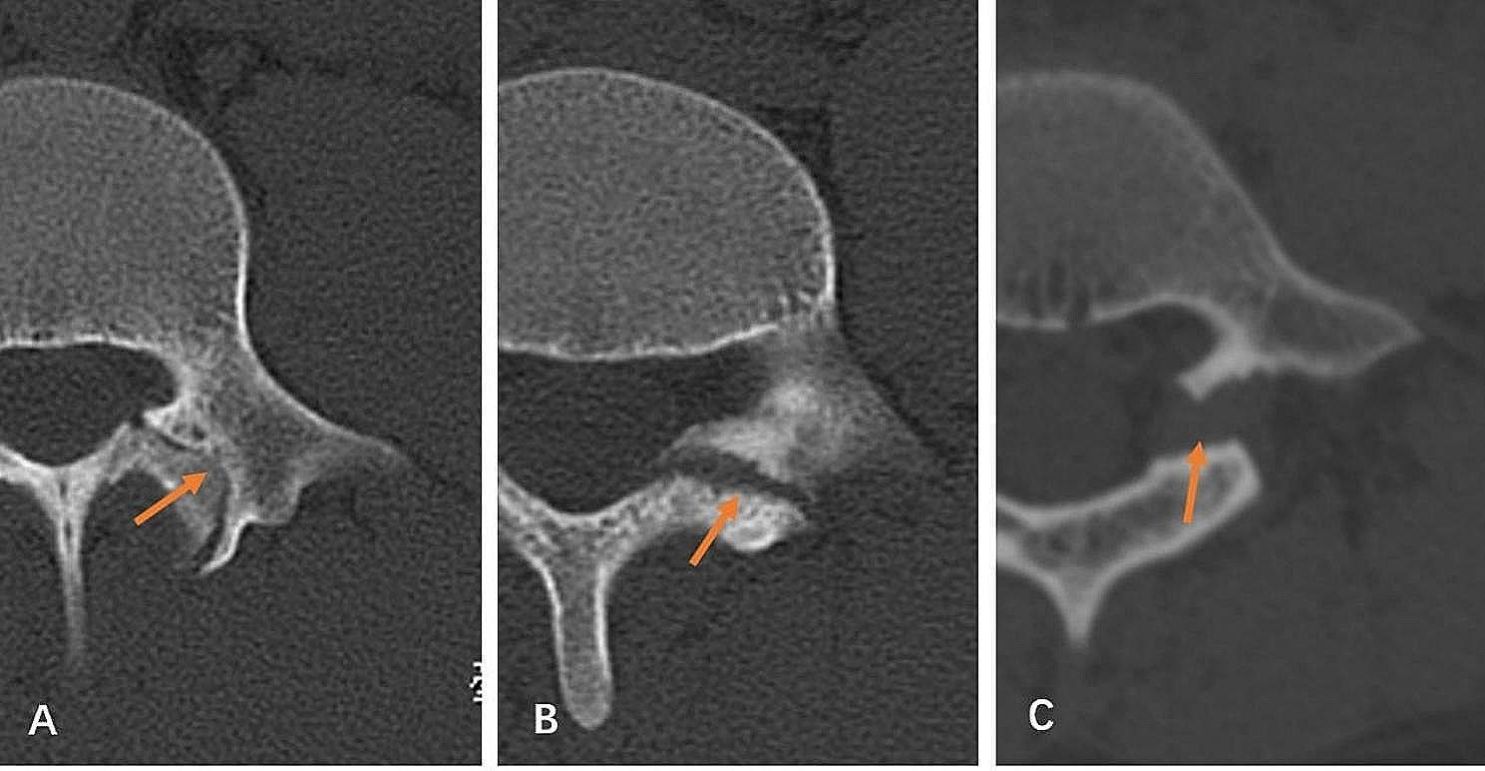
Representative CT images of different stages of pars defects (orange arrows). Stage I: Early stage, the gap is narrow, similar to hairline **(A)**. Stage II: Progressive stage, the bone gap is clear and small fragments are present **(B)**. Stage III: Terminal stage, characterized by a wide gap, atrophy, and sclerosis edge of the bony defect **(C)**

Data related to spondylolysis were recorded. This included (1) whether the spondylolysis segment was associated with spondylolisthesis, assessed by lumbar X-ray; (2) whether the spondylolysis segment was associated with spina bifida occulta (SBO); (3) the width of the defect, which was measured on reconstructed sagittal CT image in the middle of the pars interarticularis (Fig. 1A); (4) the degree of degeneration of the intervertebral disc between the spondylolysis level and the next level, evaluated by Pfirrmann’s criteria [17]. (5) the site and the angle of the defect, as reported by Fujii et al. (Figure 1B and C) [14]. (6) stage of pars defect, as reported by Fujii et al., which was evaluated by axial CT image, including: stage I - early stage, the gap was narrow, similar to the hairline; stage II - progressive stage, the bone gap was clear and small fragments were present; and stage III - terminal stage, characterized by a wide gap, atrophy, and sclerosis edge of the bony defect (Fig. 2) [14].

### Surgical technique

**Fig. 3:**
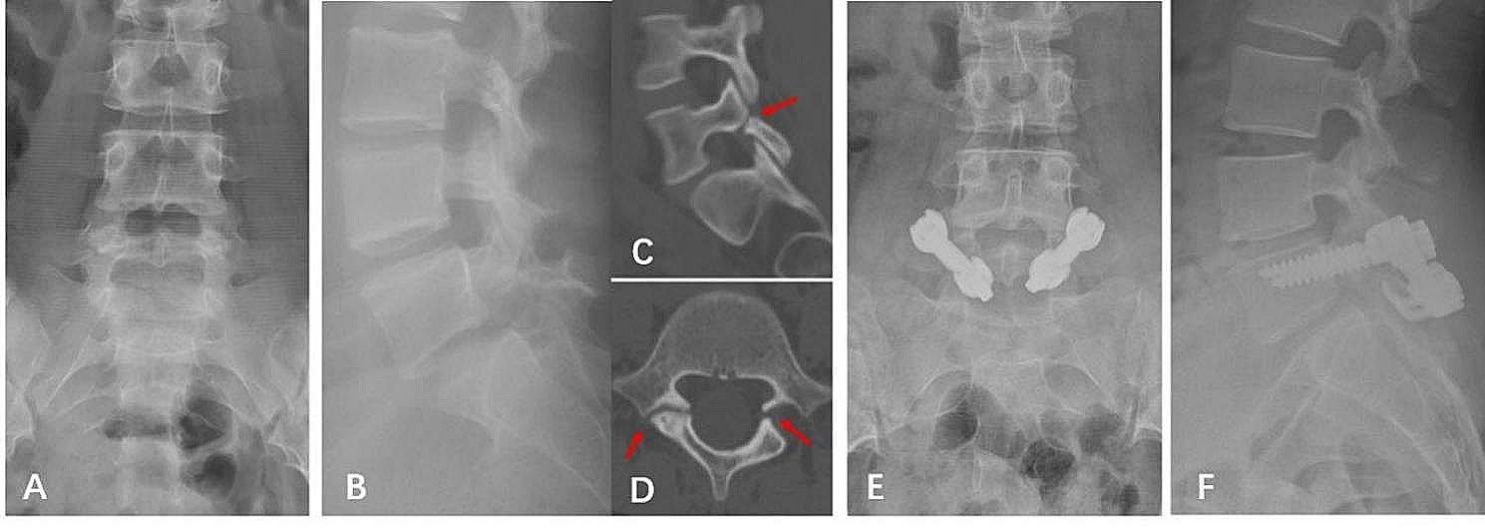
Preoperative lumbar spine X-rays of a 15-year-old female diagnosed with L5 spondylolysis **(A, B)**. Sagittal and axial CT images showing the defects in the pars interarticular **(C, D)**. Postoperative lumbar spine x-rays showing the pedicle screw- rod-laminar hook system **(E, F)**

After a standard midline approach was created, the pars defect, lamina, and entry points of pedicle screws were exposed bilaterally. Next, pedicle screws were inserted and confirmed in good position by X-rays. Then, the pars interarticularis defect was curetted to debride the fibrous nonunion and the margins of the defect were decorticated by a curet or high-speed burr. The autogenous granulated cancellous bone curetted from a small window of the iliac crest was packed into the defect and between the lamina and transverse process. Subsequently, the laminar hook was put in place and connected to the pedicle screw using a rod. Finally, the lamina hooks were compressed to close the defect and tightened (Fig. 3).

### Postoperative care

Patients were allowed to stand up and walk with a lumbar brace on the first postoperative day (POD), and discharged at POD 4 or 5. The lumbar brace was removed after 3 months, and the patient gradually returned to normal activity. All patients were followed up at 3, 6, 12 months after surgery, and then once a year.

### Bone union evaluation

**Fig. 4:**
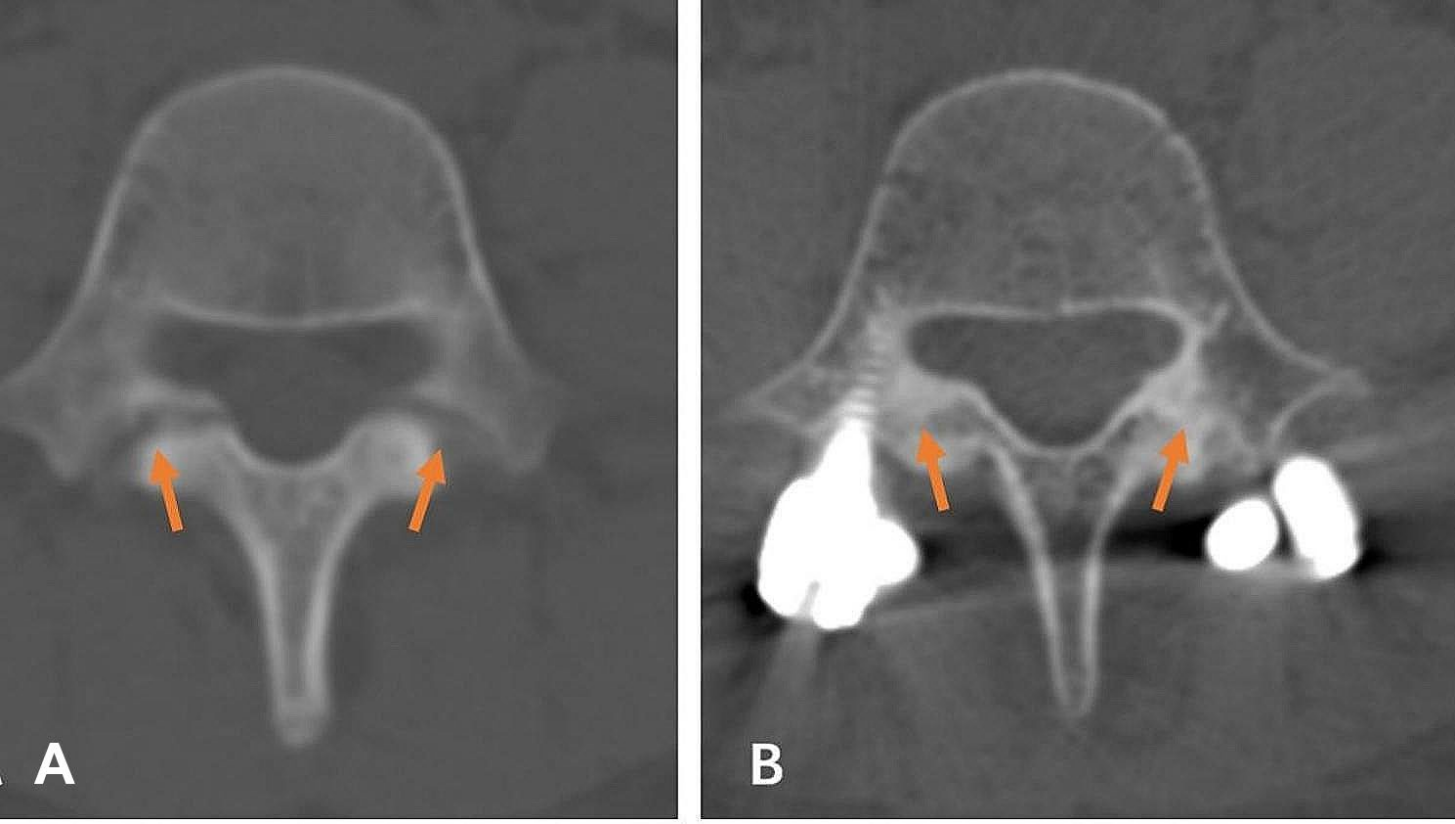
Representative axial CT images of successful fusion of the pars interarticularis defect. Pre-operative image of a patient with stage II pars defects (**A**). CT showing bilateral fusion at 14 months after pars repair surgery (**B**)

**Fig. 5:**
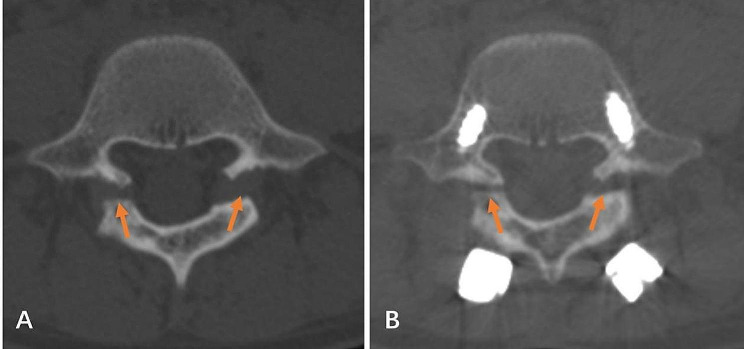
Representative axial CT images of non-fusion of spondylolysis after pars repair surgery. Pre-operative image of a patient with stage III pars defects (**A**). CT showing bilateral non-fusion at 12 months after pars repair surgery (**B**)

A CT scan was performed at one year or more to confirm whether the pars interarticularis defect had healed. Criteria for fusion of spondylolysis were a bridging bone across the defect area on axial view of last follow-up CT. Pars interarticularis was classified into a fusion and non-fusion group according to the above-mentioned criteria (Figs. 4 and 5). The patients’ state of fusion was classified as a bilateral fusion, unilateral fusion, or non-fusion. All measurements were performed by 2 well-trained orthopedic surgeons. If the data were inconsistent, a senior orthopedic surgeon was consulted.

### Clinical outcome assessment

The clinical outcomes were measured using the visual analog scale (VAS) of low back pain (LBP), the Oswestry Disability Index (ODI), and the Japanese Orthopaedic Association – 29 (JOA-29) score. When comparing clinical outcomes, unilateral fusion patients were included in the fusion group for the following reasons: (1) Despite the unilateral fusion, the lamina became integrated with the vertebral body, thereby changing from an unstable state to a stable state; (2) A 45-year follow-up natural history study demonstrated that unilateral pars defects were not associated with further spondylolisthesis or disability.18 (3) PLSH is a reliable method of fixation,2–4,6–13 thus unilateral fusion plus the biomechanical strength of PLSH may be strong enough to equate to bilateral fusion.

### Statistical analyses

Radiological classification and staging were performed independently by two observers (the first author and second author). If the two observers did not agree, final confirmation was performed by the corresponding author. The interobserver reliability was good, with kappa values of pars defect fusion, spina bifida occulta, stage of the defect, and disc degeneration degree being 0.859, 0.839, 0.813, and 0.785, respectively. Statistical data were presented as mean ± standard deviation for normally distributed data and median (first quartile, third quartile) for non-normally distributed data.

Data of the non-fusion group were compared with those of the fusion group. For normally distributed data, independent sample t-tests were used to compare between groups. The Mann–Whitney rank sum test was adopted for non-normally distributed data. The χ^2^ test was used to compare rates. Variables that were potentially associated with non-fusion on univariate analysis (*P* < 0.10) were included in the multivariable logistic regression analysis. SPSS version 21.0 (IBM Corporation, Armonk, NY, USA) was used for statistical analyses. *P* < 0.05 was considered statistically significant.

## Results

**Fig. 6:**
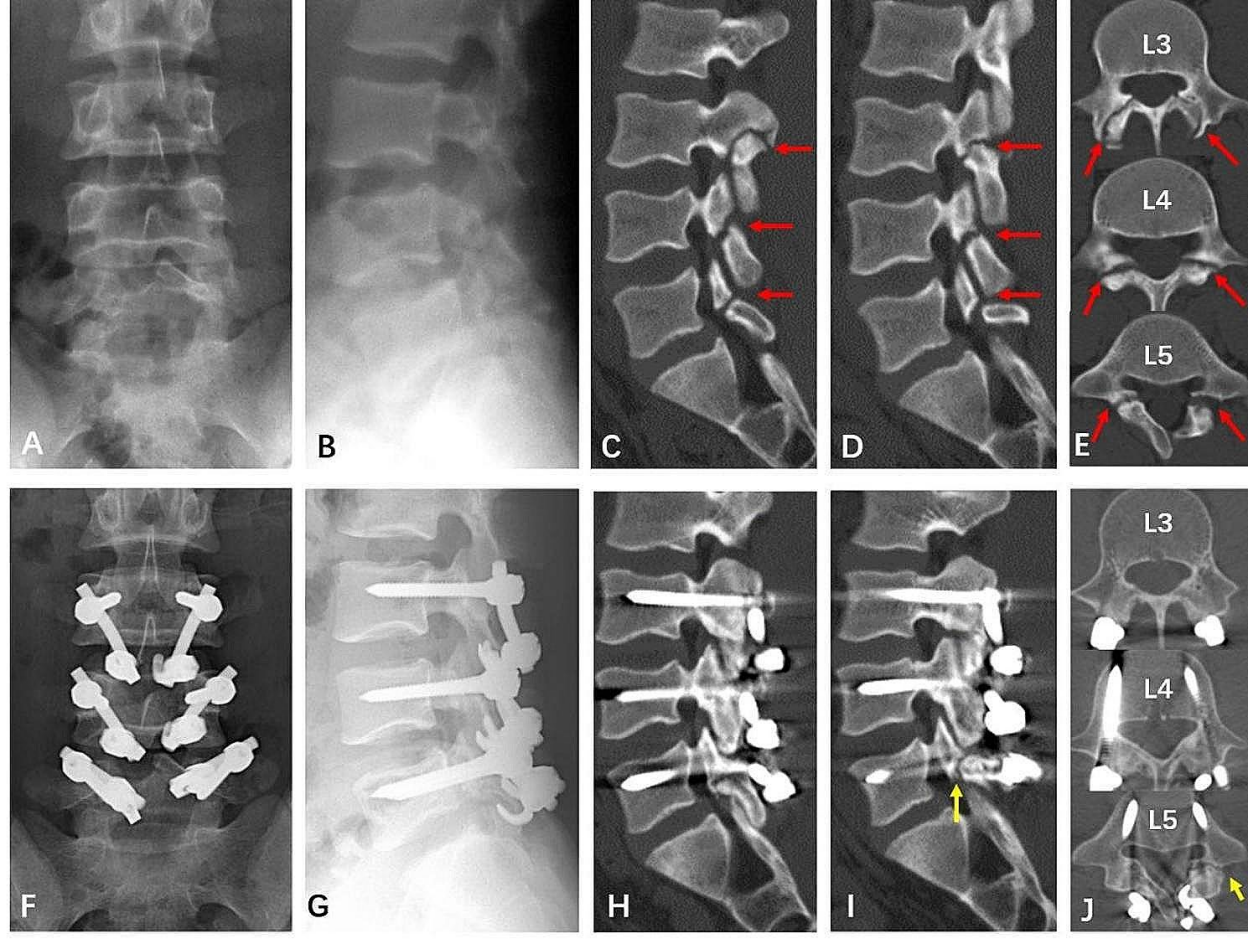
A16-year-old male, preoperative X-rays **(A, B)** and CT images **(C, D, E)** showed L3, L4 and L5 bilateral spondylolysis (red arrows in **C, D** and **E**) and L5 spina bifida occulta **(E)**. Postoperative X-rays **(F, G)** and CT images **(H, I, J)** at two-year follow-up showed union of L3, L4 and right L5 spondylolysis and non-union of left L5 spondylolysis. He had a normal daily life and no low back pain

In this study, a total of 55 adolescents and young adults with symptomatic lumbar spondylolysis with a mean age of 21.1 ± 6.3 (9–35) years were included, including 38 males and 17 females. Table 1 shows the general information and spondylolysis level distribution. Among the subjects, 42 had spondylolysis at L5, 8 had spondylolysis at L4, 1 had spondylolysis at L3, 3 had spondylolysis at both L3 and L5, and 1 had spondylolysis at L3, L4, and L5. All patients had bilateral spondylolysis and underwent direct pars repair surgery using the PSLH technique. The mean follow-up time was 24.8 ± 12.0 (12–64) months. In total, there were 120 pars defects. At the last follow-up, 101 pars defects were successfully fused and 19 were not fused as assessed by CT. The fusion rate was 84.2%. A total of 44 patients had bilateral fusion, 8 patients had bilateral non-fusion, 1 patient diagnosed with L5 spondylolysis had unilateral non-fusion, 1 patient diagnosed with L3, L4 and L5 spondylolysis had L5 unilateral non-fusion (Fig. 6), and 1 patient diagnosed with L3 and L5 spondylolysis had L5 unilateral non-fusion. In 6 patients, the spondylolysis segments were associated with SBO. Among them, 2 patients had bilateral fusion, and 2 patients had bilateral non-fusion, and 2 patients had unilateral non-fusion. The fusion rate in our cases with SBO was 50%. The fusion rates of different stages of pars defect were 100% (12/12) in stage I, 97.3% (71/73) in stage II, and 51.4% (18/35) in stage III.

**Table 1:** General information of the subjects

Age (years)		21.1 ± 6.3 (9–35)
Sex		38 males, 17 females
Spondylolysis level distribution	L3	1 patient
	L4	8 patients
	L5	42 patients
	L3 and L5	3 patients
	L3, L4 and L5	1 patient
BMI		23.8 ± 3.2
Follow-up time (months)		24.8 ± 12.0 (12–64)

The above 120 pars defects were divided into a non-fusion group (*n* = 19) and a fusion group (*n* = 101). When comparing clinical outcome-related data, patients were divided into a non-fusion group (*n* = 8, including 8 bilateral non-fusion patients) and a fusion group (*n* = 47, including the 44 bilateral fusion patients and 3 unilateral fusion patients). The results of univariate analysis are shown in Table 2.

**Table 2:** Comparison of the parameters between non-fusion group and fusion group

	Non-fusion (*n* = 19 pars interarticularis)	Fusion group (*n* = 101 pars interarticularis)	t	χ^2^	P value
Age (years)	22.0 ± 7.7	21.0 ± 6.2	0.410		0.684
Sex	Male:15	Male:69		0.861	0.354
	Female:4	Female:32			
Follow-up time (months)	18.4 ± 10.1	25.9 ± 12.0	-1.657		0.103
BMI	22.9 ± 3.7	23.9 ± 3.1	-0.841		0.404
LL(°)	54.5 ± 10.6	52.9 ± 11.3	0.370		0.713
SS(°)	41.4 ± 5.5	40.9 ± 7.2	0.154		0.878
PT(°)	10.5 ± 6.5	11.9 ± 5.8	-0.620		0.538
PI(°)	51.8 ± 8.6	52.8 ± 8.5	-0.334		0.739
Spondylolisthesis	68.4%	48.5%		2.538	0.111
SBO	31.6%	5.9%		9.005	0.003
Stage of the defect	Stage I:0%	Stage I:11.9%		39.798	< 0.001
	Stage II: 10.5%	Stage II: 70.3%			
	Stage III: 89.5%	Stage III: 17.8%			
Disc degeneration	Grade I: 5.3%	Grade I: 36.6%		11.846	0.003
	Grade II: 52.6%	Grade II: 47.5%			
	Grade III: 42.1%	Grade III: 13.9%			
Width of the defect (mm)	4.3 ± 1.8	1.9 ± 1.0	5.809		< 0.001
Site of the defect	0.54 ± 0.10	0.55 ± 0.14	-0.573		0.568
Angle of the defect (°)	16.5 ± 12.4	15.6 ± 11.9	0.290		0.773

Variables that were positive (*P* < 0.10) in the univariate analysis were included in the multivariable logistic regression analysis. Factors that correlated with non-fusion after direct pars repair surgery were: whether the spondylolysis segment was associated with SBO (*P* = 0.031), stage of the defect (*P* = 0.047), width of the defect (*P* = 0.001), and disc degeneration (*P* = 0.014) (Table 3).

**Table 3:** Logistic regression analysis of the factors associated with non-fusion

	B	Odds Ratio (OR)	95% confidence interval (CI) for OR	P value
SBO	-2.338	0.092	0.010 ~ 0.805	0.031
Stage of the defect	-1.857	0.156	0.025 ~ 0.975	0.047
Width of the defect	-0.931	0.394	0.218 ~ 0.713	0.002
Disc degeneration	-1.775	0.170	0.041 ~ 0.702	0.014

B stands for regression coefficient

There were no significant differences in the pre-operative VAS score, ODI and JOA-29 score between the two groups (Table 4). However, non-fusion patients seem to have a trend of worse clinal outcomes, with poorer post-operative ODI (*P* = 0.083) and JOA-29 scores (*P* = 0.022) (Table 4). Among the 8 non-fusion patients, 1 patient underwent L5/S1 interbody fusion surgery because of worsening low back pain and spondylolisthesis 3 years after pars repair surgery, 1 patient had screw loosening and intermittent moderate low back pain, and 3 cases had no improvement in low back pain after surgery and were not satisfied with the outcome. Of the 47 fusion patients, 1 patient had poor wound healing, 1 patient had iliac grafting donor site pain (VAS = 3), and 1 patient had pedicle screw malposition without symptoms. Six patients had their internal fixations removed after fusion on their own request.

**Table 4:** Comparison of clinical outcomes between non-fusion and fusion groups

	Non-fusion (*n* = 8 patients)	Fusion group (*n* = 47patients)	t	z	P value
Pre-op VAS	4.1 ± 2.0	5.3 ± 1.6	-1.831		0.073
Pre-op ODI (%)	16.8 ± 12.7	23.9 ± 12.9	-1.460		0.150
Pre-op JOA-29	24.8 ± 3.3	22.7 ± 3.9	1.400		0.167
Post-op VAS	2.0 (1.0, 4.5)^a^	1.0 (1.0, 2.0)^b^		-1.603	0.109
Post-op ODI (%)	9.4 (2.5, 18.9)^a^	4.0(2.0, 6.7)^b^		-1.736	0.083
Post-op JOA-29	25.5 (22.3, 28.0)^a^	28.0 (27.0, 29.0)^b^		-2.282	0.022

^a^ No significant difference (*P* > 0.05) compared with correlated pre-op data

^b^ Significant difference (*P* < 0.05) compared with correlated pre-op data

## Discussion

Since Buck first reported the application of internal fixation for pars repair in 1970, a variety of internal fixation methods have been presented, such as the Scott technique, Morscher technique, PLSH technique, etc. [2, 6]. Techniques with more rigid modes of fixation tended to have better fusion rates for the pars defect [6, 19]. The PLSH technique provides a stronger fixation with a pedicle screw and a more compression force with a laminar hook connected by a rod. The results of our study showed that the fusion rate of the PSLH technique at follow-up of at least 1 year was 84.2%, which was consistent with the results reported by Zayan et al. [12], but a bit lower than the pooled average rate (90.2%) shown by the meta-analysis by Mohammed et al. [6]. There may be several reasons for these differences. First, there should be some factors associated with non-fusion in this cohort of patients, which were explored in this study. Second, criteria for fusion were more stringent with helical CT at high resolution compared to X-ray. Several studies reported that the CT-assessed fusion rate was obviously lower than the radiograph-assessed fusion rate, thus indicating that the non-fusion might be underdiagnosed as fusion because of low resolution of the radiograph in previous studies [20–22]. Our study showed that SBO, a terminal stage of the defect, a wider defect gap, and higher disc degeneration degree (Pfirrmann’s grade III) were independent factors associated with non-fusion after pars repair using PSLH.

There are few reports that focus on surgical management of spondylolysis with SBO. Yamamoto et al. reported segmental wire fixation for spondylolysis associated with SBO, two of four (50%) SBO cases showed non-fusion bilaterally after an average of 32 months of follow-up [23]. Zhang et al. reported a lower healing rate in patients with SBO compared to patients without SBO when the spondylolysis was treated with intersegmental pedicle screw fixation [24]. The fusion rate in our cases with SBO was 50%, which supported that SBO is one of the factors associated with non-fusion after pars repair surgery. The underlying mechanism of action by which SBO affects healing may be: (1) the vertebrae with SBO are mostly accompanied by smaller lamina or lamina dysplasia, which cannot provide strong anchoring points for internal fixation; (2) the pedicle screws, laminar hooks, and laminae are in one piece in a normal vertebrae after pars repair by the PSLH technique, while in a vertebrae with SBO the left and right laminae are separated, so that the pedicle screw-hook-laminae complex on both sides are also separated, resulting in less reliable fixation compared to that of normal laminae.

The data obtained in this study showed that a terminal stage of defect was related to non-fusion after pars repair surgery by the PSLH technique, which was consistent with the results of conservative treatment [5, 15, 16]. A terminal stage often suggests that the defects have atrophy and sclerosis edges, which are not conducive to the bony healing. Morita et al. reported a healing rate of 0% in the terminal stage after conservative treatment, while a healing rate of 73% was observed in the early stage and 38.5% in the progressive stage [16]. In our study, the fusion rate of a terminal stage pars defect was significantly lower compared to that of early stage and progressive stage. Therefore, more attention should be paid to decortication of the sclerosis edge and bone grafting during surgery.

The width of the defect is also one of the factors affecting bone healing after pars repair. This is not hard to understand, because theoretically, the wider the gap, the more difficult it is to form a continuous bony connection. Berger et al. suggested that a good candidate for direct repair has no more than a 2-mm gap at the pars interarticularis [2]. Moreover, Debnath et al. believed that a gap of less than 4 mm was one of the main factors that may predict a successful surgical outcome [3]. In the study by Roca et al., all patients had a gap of less than 3 mm [9]. Our results showed that the average gap in the non-fusion group was significantly wider than that of the fusion group (4.3 ± 1.9 mm vs. 1.9 ± 1.0 mm). However, the accurate cut-off for the defect gap requires additional studies with a larger sample size. A wider gap indicated more slippage of the vertebra and a terminal stage of spondylolysis, and these factors will increase the risk of non-fusion after pars repair.

There is still controversy about the effect of the disc degeneration on pars repair surgery. On the one hand, it is believed that the best candidate for pars repair should be no disc degeneration, while on the other hand, it is believed that mild to moderate (up to grade III) disc degeneration is acceptable [2, 3, 25]. In a systemic review, Kumar et al. concluded that grade III/IV disc degeneration was one of the causal factors for negative long-term outcomes after repair surgery [25]. Our findings support that moderate disc degeneration (grade III) is a factor associated with nonunion. As for the mechanism, it is still unclear, and no relevant studies have been conducted before. Disc degeneration may be related to various factors that are not conducive to defect union, thus indirectly affecting fusion rates: (1) degenerated disc in is more likely to be associated with instability or spondylolisthesis in spondylolysis patients, which is not conducive to the fusion of pars defects. (2) Patients with moderate and severe disc degeneration may have a longer course of disease, so the defects are more likely to be in the terminal stage and had a large gap, which also makes the fusion more difficult. However, larger sample size studies are still needed to verify the relationship between disc degeneration and fusion of the defects.

The relationship between fusion or not and clinical outcomes is still unclear. Some studies highlight that there may be no correlation or poor correlation between radiological/CT-based fusion and clinical outcomes [22, 26]. Our study and some other studies considered that complete fusion may confer stability at the pars defect, and that non-fusion may be related to relatively poor clinical results [11, 20, 21]. Lee et al. reported a solid fusion (evaluated by CT scan) at the pars defect in only 50% of the patients treated by the Buck’s method, which was significantly lower than the fusion rate presented in other studies [26]. They found whether the fusion was achieved did not relate to the clinical outcomes. Hioki et al. reported that the average improvement rate was 78.9% in the bilateral fusion group, 63.6% in the unilateral fusion group, and 29.8% in the non-fusion group, and the differences among the 3 groups were significant [20]. Notably, in our study, 3 out of 8 patients with a bilateral non-fusion had no relief of low back pain after surgery. More studies with a prospective design may be needed to determine the relationship between non-fusion and clinical outcomes.

To our knowledge, this is the first study that focuses on the potential risk factors for non-fusion after pars repair surgery. This study has several limitations. First, it has the inherent limitations of a retrospective study and a relatively low follow-up rate (70.5%), which may cause possible confounding factors and bias. Second, this study has a small sample size. A small sample size of non-fusion group may affect the reliability of statistics, especially in multivariable logistic regression analysis. However, this does not mean that our results are without value. The results are consistent with our clinical findings. This is an exploratory study, and future studies with larger sample sizes are needed to verify these results. Furthermore, for patients with a non-fusion at the last follow-up, the follow up time may not be long enough. By increasing the follow-up time, there is a possibility of healing in these patients.

## Conclusion

Direct pars repair by PSLH is a reliable treatment for lumbar spondylolysis with a fusion rate of 84.2%. The data obtained in the present study suggests that association with SBO of the spondylolysis segment, a terminal stage of the defect, a wider defect gap, and grade III disc degeneration may be factors associated with non-fusion after direct pars repair of lumbar spondylolysis with PLSH. Non-fusion patients after pars repair appear to have worse clinical results than fusion patients.

## Data Availability

The datasets used and analyzed during the current study are available from the corresponding author on reasonable request.

## Abbreviations

PSLH: Pedicle screw and lamina hook
CT: Computerized tomography
LL: Lumbar lordosis
PI: Pelvic incidence
PT: Pelvic tilt
SS: Sacral slope
SBO: Spina bifida occulta
POD: Postoperative day
VAS: Visual analog scale
LBP: Low back pain
ODI: Oswestry disability index
JOA: Japanese Orthopaedic Association
